# Can Selective MHC Downregulation Explain the Specificity and Genetic Diversity of NK Cell Receptors?

**DOI:** 10.3389/fimmu.2015.00311

**Published:** 2015-06-16

**Authors:** Paola Carrillo-Bustamante, Can Kesmir, Rob J. de Boer

**Affiliations:** ^1^Theoretical Biology and Bioinformatics, Department of Biology, Utrecht University, Utrecht, Netherlands

**Keywords:** agent-based modeling, NK cells, immunoevasion, evolution, NK cell receptors

## Abstract

Natural killer (NK) cells express inhibiting receptors (iNKRs), which specifically bind MHC-I molecules on the surface of healthy cells. When the expression of MHC-I on the cell surface decreases, which might occur during certain viral infections and cancer, iNKRs lose inhibiting signals and the infected cells become target for NK cell activation (missing-self detection). Although the detection of MHC-I deficient cells can be achieved by conserved receptor-ligand interactions, several iNKRs are encoded by gene families with a remarkable genetic diversity, containing many haplotypes varying in gene content and allelic polymorphism. So far, the biological function of this expansion within the NKR cluster has remained poorly understood. Here, we investigate whether the evolution of diverse iNKRs genes can be driven by a specific viral immunoevasive mechanism: *selective MHC downregulation*. Several viruses, including EBV, CMV, and HIV, decrease the expression of MHC-I to escape from T cell responses. This downregulation does not always affect all MHC loci in the same way, as viruses target particular MHC molecules. To study the selection pressure of selective MHC downregulation on iNKRs, we have developed an agent-based model simulating an evolutionary scenario of hosts infected with herpes-like viruses, which are able to selectively downregulate the expression of MHC-I molecules on the cell surface. We show that iNKRs evolve specificity and, depending on the similarity of MHC alleles within each locus and the differences between the loci, they can specialize to a particular MHC-I locus. The easier it is to classify an MHC allele to its locus, the lower the required diversity of the NKRs. Thus, the diversification of the iNKR cluster depends on the locus specific MHC structure.

## Introduction

Natural killer (NK) cells are key players of the innate immune system that eliminate viral infected and tumor cells. To detect aberrant cells and remain tolerant to healthy tissue, NK cells have several inhibiting and activating receptors, tightly regulating their cytotoxicity. Crucial for this NK cell mediated immune recognition is the detection of major histocompatibility complex (MHC) class I molecules on the surface of target cells. Because several MHC molecules are ligands for inhibitory NK cell receptors (iNKRs), they signal NK cells to remain silent to self healthy cells. However, the expression of MHC molecules decreases during some viral infections and cancer, resulting in reduced inhibiting signals for the NK cell, and hence in NK cell activation [missing-self detection (1)].

Some of the NK cell receptors (NKRs) are encoded by a variety of polymorphic multigene families, including the killer immunoglobulin-like receptors (KIRs) in higher primates (2, 3), the CD94/NKG2 receptor family in lemurs (4), and the Ly49 gene family in rodents and equids (5, 6). These polygenic NKR families encode haplotypes that differ in gene content and allelic polymorphism, resulting in a large variation on the population level. Because NKR genes segregate independently from MHC-I genes, the number of possible receptor-ligand combinations is immense. In addition to these diverse receptor families, there are also conserved NKR genes, e.g., the inhibiting CD94/NKG2A receptors in humans and mice. These inhibiting receptors interact with the also monomorphic HLA-E in humans and Qa-1*^b^* in mice (7–10), providing a simple system for the recognition of missing-self. But, if these conserved receptor-ligand interactions are able to successfully detect missing-self, why have other NKR families evolved to become so polymorphic, polygenic, and specific?

Because of the evolutionary arms race between infectious pathogens and the immune system, one plausible explanation for the diversification of NKRs is the selection pressure on NK cells imposed by the successful immunoevasins evolved by several pathogens (11, 12). One of the best studied escape mechanisms is that of MHC-I mimics encoded by Cytomegalovirus (CMV) in mice and humans (11, 12). To escape from T cell responses, CMV downregulates the expression of MHC-I and additionally encodes MHC-I decoys to evade missing-self detection by NK cells (12, 13). We have previously addressed the effect of decoy molecules on the evolution of NKRs with agent-based models (ABM) (14, 15), showing that decoy evolving viruses exert a strong selection pressure on NKRs, resulting in a diversified repertoire of specific iNKRs.

Importantly, our previous studies also showed that for simple detection of MHC downregulation, degenerate iNKRs are advantageous (14). Therefore, neither specificity nor diversity evolves in populations infected with viruses downregulating MHC molecules. In those simulations, we assumed that *all* MHC molecules in the host were targeted by the virus. However, a more common immunoevasive mechanism is that of *selective MHC downregulation*, i.e., when the downregulation does not affect all MHC molecules in the same way, but only particular MHC-I molecules are targeted by the virus. Several viruses, including Epstein-Barr-Virus (EBV), CMV, and the human immunodeficiency virus (HIV), decrease the expression of MHC-I of the cells they infect in a selective manner [reviewed in Ref. (16)]. For example, HCMV encodes several immunoevasin proteins that selectively downregulate the expression of MHC-I on the cell surface (16) like US2 and US11, each targeting particular HLA-A and HLA-B alleles by promoting their export into the cytosol for proteosomal degradation (17–19). In addition to selective MHC-downregulation, HCMV encodes proteins that enhance MHC-I expression to inhibit NK cells, such as UL40 having a high sequence similarity to protein fragments of HLA-C alleles (20, 21). HIV-1 also decreases the expression of particular HLA alleles. HIV Nef binds to the cytoplasmic tails of the HLA-A and HLA-B molecules in the ER, re-directing them to endolysosomal compartments for degradation (22). By contrast, HLA-C and -E have slightly different cytoplasmic tails, so that Nef no longer hampers their transport to the cell surface. This smart strategy prevents HIV-infected cells to by lysed by NK cells (23–25).

Since the HLA molecules presenting peptides to T cells (HLA-A and -B) tend to be downregulated while those inhibiting NK cells (HLA-C and -E) remain expressed, selective MHC downregulation seems to be a good viral strategy to avoid missing-self detection. This selective downregulation can in turn shape the evolution of the NK cell receptors, as specific inhibiting receptors that exclusively recognize only a subset of MHC molecules can be advantageous to detect missing-self.

Our previous studies showed that the expression of MHC-I mimics by viruses provides a solid explanation for the diversification of iNKRs. Here, we investigate whether selective MHC downregulation can exert sufficient selection pressure to drive the evolution of a polygenic and polymorphic NKR system in the absence of decoys. We develop an ABM of host populations infected with herpes-like viruses causing chronic infections. Our simulations show that NKRs readily evolve specificity for a single MHC locus. The evolution of these “MHC locus specific” detectors depends on the similarity of the MHC molecules within each locus, and the difference between MHC loci. Accordingly, there is selection for polygenicity and polymorphism only in those populations having MHC-I molecules that differ sufficiently within one locus and between the loci.

## Results

### Agent based model

To study the evolution of NKRs in a host population, we developed an ABM similar to our previously described models (14, 15). Briefly, the host population consists of simplified humans infected with non-lethal viruses causing chronic infections. The hosts are diploid, carrying two polymorphic MHC loci and an NKR cluster in different chromosomes. We only model inhibitory NKRs (iNKRs) in this work, as only iNKRs are involved in missing-self detection. iNKRs and MHC molecules are modeled with random sequences of 0s and 1s (i.e., bit strings) as a simplified representation of amino acids. Whenever the longest adjacent complementary match between two strings exceeds a binding threshold L, the molecules can interact (Figure S1 in Supplementary Material). We model two different groups of MHC molecules, henceforth referred to as MHC-X and MHC-Y. The molecules within each MHC group are somewhat similar to each other, and we vary the hamming distance (HD) of the molecules within one group between 2, 4, 6, and 8 (see Materials and Methods).

Initially, all hosts carry the same randomly generated NKR cluster, which is composed of one gene (i.e., we initialize the populations with homozygous individuals). This iNKR is degenerate, i.e., it can recognize every MHC molecule in the population. Upon birth, individuals inherit one NKR haplotype from each parent. During this process, NKRs can mutate their sequence and their binding threshold L (Figure S1 in Supplementary Material), allowing for the emergence of novel receptors (see Materials and Methods). If a newly generated receptor is so specific that it fails to recognize any MHC molecule in the population, it will be called a *pseudogene*. The maximal number of NKRs that we allow per host is five genes per haplotype in these simulations. We focus on the evolution of NKRs, and therefore fix the polymorphism of the MHC-X and -Y molecules throughout the simulations.

During development, NK cells undergo an education process during which their reactivity is “tuned.” The general consensus is that the binding of iNKRs with their cognate MHC molecules renders these NK cells functional capacity (26–28). We implement this process at birth by evaluating the binding of iNKRs with their MHC ligands in the host. For simplicity, we do not model individual NK cell subsets, but estimate the total repertoire of “licensed” receptors per host. In each host, the repertoire of licensed receptors consists of all iNKRs binding at least one of the host’s MHC molecules. In our model, only licensed NKRs participate during an immune response, assuming that at least one NK cell subset will express at least one licensed receptor and will become activated and expand upon infection.

We model two viral species causing chronic infections (viral species A and B). Both viral species can escape the cytotoxicity of T cells by downregulating the expression of the MHC I molecules on the surface of the infected cells. The downregulation is specific to each locus, i.e., virus species A downregulates the MHC-X molecules in the host, while virus B downregulates the host’s MHC-Y molecules.

Upon transmission, the host will enter a phase of acute infection, after which it can either recover or become chronically infected. Individuals clearing an infection become immune against that particular viral species for a period of 10 years. Hosts can be co-infected with both viral species.

**Figure 1:**
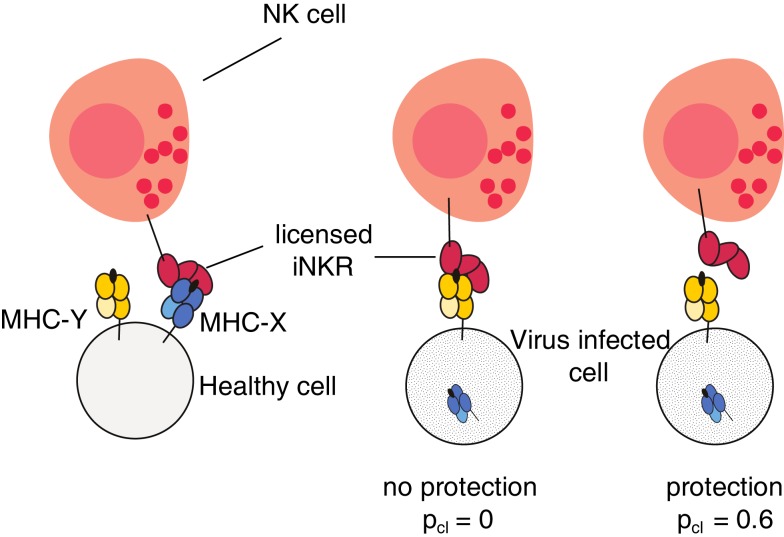
**Model cartoon of the protection after infection with an MHC-downregulating virus**. An iNKR becomes licensed if it binds at least one MHC molecule in the host. In this example, the iNKR is licensed by binding the MHC-X molecule in that host. The virus downregulates the expression of all MHC-X molecules. The infection can be cleared with a probability p_cl_ = 0.6 if and only if the licensed iNKR fails to bind all MHC-Y molecules in that host (see Materials and Methods).

The probability of clearing the infection depends on the interactions between the iNKRs and the expressed MHC-I molecules in the host. For a virus to be cleared, the licensed repertoire of iNKRs must be able to detect MHC downregulation. If a virus downregulates the MHC-X molecules, and all licensed iNKRs interact only with the MHC-Y molecules, the NK cells will continue to be inhibited, and will not detect the altered MHC expression. But if at least one licensed iNKR binds *none* of the expressed MHC-Y molecules, the NK cells carrying that receptor will lose an inhibiting signal when MHC-X is downregulated, become activated, and provide protection (illustrated in Figure 1).

**Figure 2:**
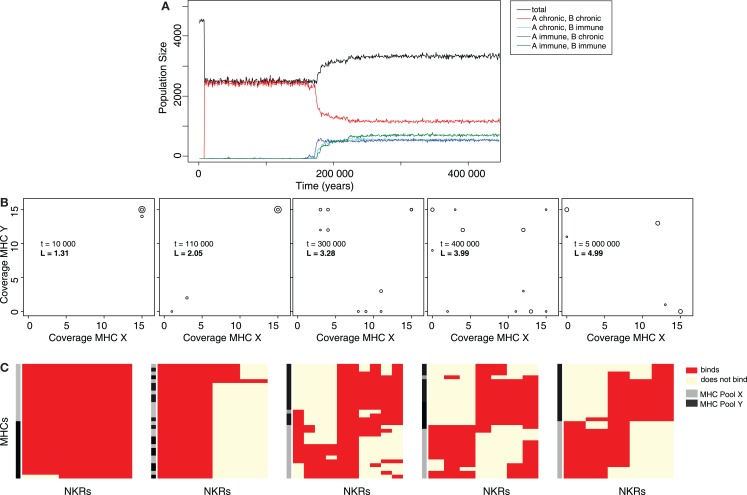
**The evolution of MHC-X and MHC-Y detectors provides immunity**. **(A)** Representative simulation of a host population infected with both viral species. After their introduction at t_1_ = 10000, the viruses spread rapidly through the population, infecting most individuals, and causing a drastic decrease of the total population size (black line). Most hosts are chronically infected with both viruses simultaneously (red line). After several host generations, some hosts evolve immunity to viruses downregulating MHC-X molecules but remain chronically infected with viruses downregulating MHC-Y (blue line). Shortly afterwards, other individuals become immune to viruses downregulating MHC-Y molecules while being chronically infected with viruses downregulating MHC-X (cyan line). Finally, hosts that are able to clear both viruses evolve (green line), resulting in a recovery of the population. **(B)** MHC coverage per iNKR and average binding threshold (L) at different time points (snapshots taken from the video provided in the Supplementary Material). Each iNKR is depicted by one circle, the size of which represents its frequency in the population. The position of each circle shows how many MHC-X and MHC-Y molecules in the population each iNKR can recognize. **(C)** Clusters of iNKRs at different time points according to the “current” iNKR-MHC-I binding matrix. We cluster both rows (MHC molecules) and columns (iNKRs), by using the Manhattan measure of distance and the Ward clustering algorithm. The heat map colors represent the iNKR-MHC-I binding; red illustrating binding and yellow no binding. The MHC molecules in this simulation were modeled with a hamming distance (HD) of two.

In a typical simulation, both viral species are introduced after the population has stabilized (t_1_ = 10000 years). Very rapidly, the viruses spread through the population, infecting almost every individual, and causing a drastic reduction of the population size (Figure 2A, black line). After approximately 2000 host generations, individuals that can become immune to each or both viral strains evolve (Figure 2A, cyan, blue, and green lines), causing a rapid recovery of the total population size. These results suggest that the initially susceptible host population evolves an NKR system, providing immunity to both types of infections.

### Specific iNKRs protect hosts from (viral) infections

To study how the evolution of iNKRs allows the host population to recover, we next analyze the iNKRs before and after a long evolutionary period (Figure 2B). The initial haplotype composed of one degenerate iNKR recognizes all MHC-I in the population, covering the entire space of both MHC-X and -Y molecules. After the introduction of the viruses, there is selection for more specific iNKRs, as shown by the increase of their binding threshold L (Figure 2B). As more specific iNKRs evolve, the number of immune individuals increases, indicating that the expansion of these specific iNKRs is required for the hosts to clear the infection.

Because of their higher specificity, i.e., higher L, the evolved iNKRs are expected to recognize fewer MHC molecules than the initial degenerate receptor. Interestingly, some iNKRs are still able to recognize most of the MHC-I molecules within one locus, despite their higher specificity (Figure 2B). For example, at t_end_ = 5000000, there is one iNKR recognizing all 15 MHC-X molecules, while binding none of the MHC-Y molecules. This specialization to the MHC-I loci allows the host to successfully detect the locus specific MHC-downregulation.

In our model, two processes are crucial in determining NK cell mediated protection. First, an iNKR must be licensed to participate in an immune response. Second, the licensed receptor should be able to detect missing-self. For an iNKR to become licensed, it must recognize as many MHC molecules as possible in the population. For instance, an iNKR binding all MHC-X molecules in the population will get licensed in every host. However, during an infection with a virus downregulating MHC-X, the licensed iNKR will only detect missing-self if it fails to recognize all MHC-Y molecules. Therefore, an “MHC-X detector” (i.e., an iNKR recognizing more MHC-X than Y molecules) is protective against viruses downregulating MHC-X molecules. Similarly, an MHC-Y detector is protective against MHC-Y downregulating viruses.

We further study the evolution of MHC-X and MHC-Y detectors by clustering the iNKRs according to their binding to all MHC-I molecules in the population (Figure 2C). Our analysis reveals that after a long evolutionary period most iNKRs specialize to one MHC locus, having little overlap in the detection of MHC-X and MHC-Y molecules. Taken together, our results show that viruses downregulating MHC molecules selectively drive the evolution of iNKRs that are specific for different MHC-I loci.

### Specialization to the MHC-I groups depends on the similarity of the MHC molecules

So far, we analyzed results from simulations where MHC molecules were highly similar (i.e., MHC molecules having HD = 2). To analyze how our results depend on the similarity of the MHC molecules within each locus, we perform several simulations with a HD of 4, 6, and 8. Additionally, we carry out one set of simulations in which all MHC molecules were random bit strings.

**Figure 3:**
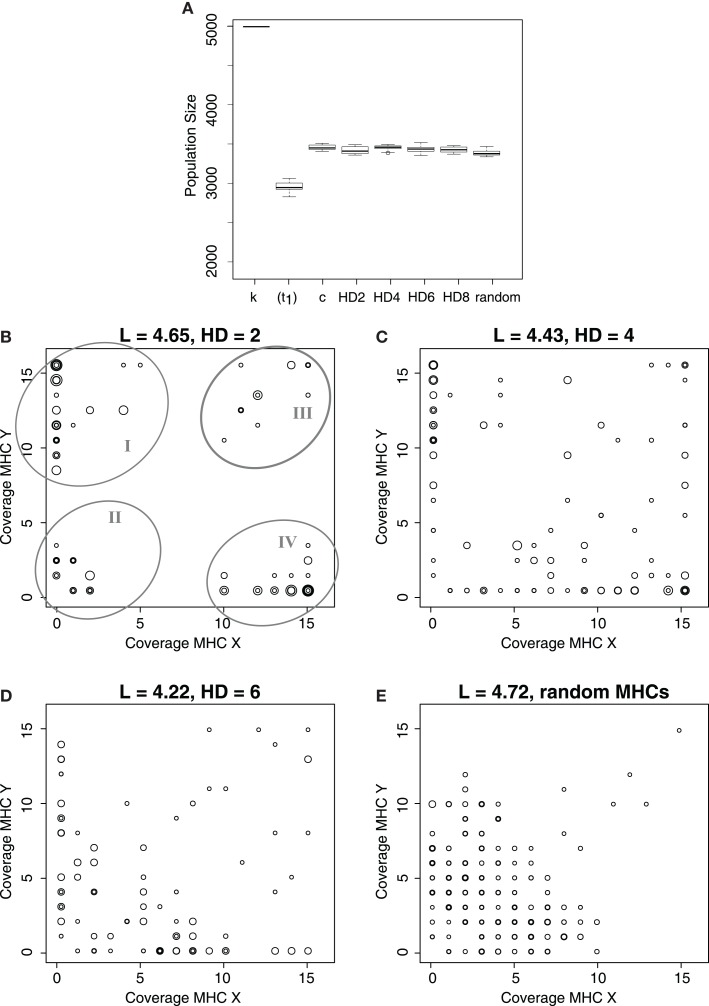
**Evolution of MHC-X and MHC-Y detectors depends on the similarity of the MHC molecules**. **(A)** depicts the average population size out of 10 simulations at the carrying capacity (k), immediately after the infection, i.e., t_1_ = 10000 (for HD 2), and at t_end_, for simulations considering different similarity in the MHC molecules. As a control, c, we simulate populations infected with both viruses, where the probability of clearing the infection is maximal (i.e., p_cl_ = 0.6) does not depend on the iNKR-MHC interactions. After a long evolutionary period, all populations evolve immunity. **(B–E)** show the MHC coverage per iNKR and the average binding threshold (L) at t_end_ out of all 10 simulations. Each host population has a different degree of MHC-I similarity, with HD = 2 **(C)**, HD = 4 **(C)**, HD = 6 **(D)**, and completely random MHC molecules **(E)**. Each iNKR is depicted by one circle, the size of which represents its frequency in the population. The position of each circle shows how many MHC-X and MHC-Y molecules in the population each iNKR can recognize. The boxes in **(A)** represent the interquartile range, and the bold horizontal lines represent the median taken out of 10 simulations.

Regardless of the similarity of the MHC molecules, the initially susceptible host populations recover from the infection after a long evolutionary period (Figure 3A). Similarly, although all host populations evolve more specific iNKRs irrespective of the HD, the evolution of specific MHC detectors is more difficult as the similarity of the MHC molecules within one locus is decreased (Figures 3B–E). Host populations carrying the most similar MHC-I molecules (i.e., HD = 2) evolve four distinct types of iNKRs: a large group of MHC-Y detectors (ellipse I in Figure 3B), a small group of highly specific receptors (recognizing few MHC-X and -Y molecules, ellipse II), a small group of degenerate receptors (ellipse III), and a large group of MHC-X detectors (ellipse IV). With decreasing MHC similarity (i.e., increasing HD among MHC molecules), the classification between the groups is less clear (Figures 3C,D), and when considering random MHCs there is hardly any distinction between of X or Y detectors (Figure 3E). In populations without any locus specific MHC structure, the majority of iNKRs evolve high specificity, and bind therefore only a few molecules of each group. Additionally, there is purifying selection in these populations, as some MHC-X and -Y detectors evolve, despite the low similarity of their ligands. However, the evolution of “excellent” X or Y detectors (i.e., iNKRs recognizing all MHC alleles from X without binding any molecule from the Y locus, and vice versa) can only occur if the MHC alleles differ sufficiently between the loci.

These results were consistent for all 10 simulations we performed, showing that iNKRs become specific as a result of the selection pressure imposed by the virus. However, the specialization to the MHC loci occurs mainly if the latter have a structure that the iNKRs can adapt to (compare Figure 3E with Figures 3B–D).

In all these simulations, the host populations evolve a similar binding threshold, L, regardless of the similarity among their MHC-I molecules (Figure 3). This is a surprising observation because it indicates that the binding threshold is not the only factor mediating specificity and specialization of iNKRs to particular MHC molecules. To analyze the NKR specificity further, we next determine the complementarity of iNKRs to the MHC-X and MHC-Y molecules.

**Figure 4:**
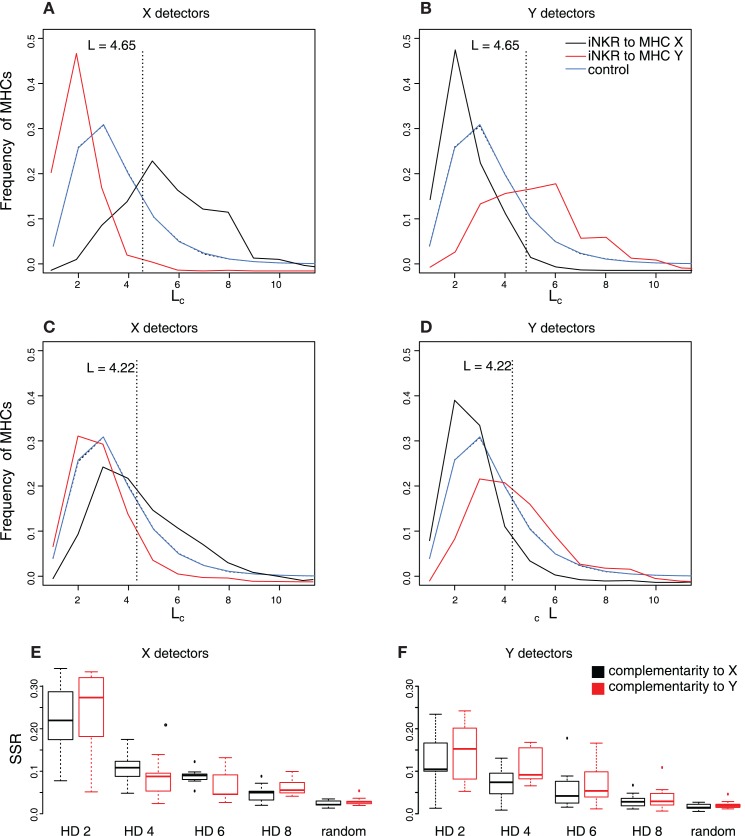
**Evolution of NKR binding affinity depends on the similarity of the MHC molecules**. We study the evolution of the binding affinity by comparing the complementarity of iNKRs to their MHC-I molecules. **(A–D)** depict the fraction of MHC molecules that are recognized with a particular complementary adjacent length L_c_. The blue curve describes the expected frequency distribution determined by measuring the L_c_ between 1000 random NKRs and a pool of random MHC molecules, and repeating this experiment 1000 times. The black and red curves represent the frequency distribution of the evolved iNKRs to MHC-X and MHC-Y molecules, respectively, at t_end_. The frequency distribution was determined by measuring the L_c_ of the evolved MHC-X detectors **(A,C)** and MHC-Y detectors **(B,D)** to the MHC-X and MHC-Y molecules in their own populations, and averaging over 10 simulations. **(A–B)** show simulations considering similar MHC molecules (i.e., HD = 2), and **(C–D)** show simulations with less similar MHC molecules (i.e., HD = 6). The sum of squared residuals (SSR) from the observed complementarity distributions to the expected L_c_ distribution is summarized for X detectors **(E)** and Y detectors **(F)** for all 10 simulations performed per setting. The boxes represent the interquartile range and the bold horizontal lines represent the median taken out of 10 simulations.

To have an expectation for the complementarity between iNKRs and MHC molecules, we generate a thousand random iNKRs and measure their longest complementary adjacent match (L_c_) to randomly generated MHC molecules (see Materials and Methods and the blue lines in Figures 4A–C). This analysis reveals that a random NKR is expected to bind 30% of random MHC molecules with a maximal complementary adjacent match of 3 bits, and no more than 5% of random MHC molecules with a maximal adjacent complementary match of 6 bits. We use this “expected” distribution as the control case. We next determine the distributions of the *evolved* iNKRs in our simulations by measuring the longest complementary adjacent match between each evolved X and Y detectors and the MHC-X and -Y molecules in the same population. The frequency distributions of L_c_ between the evolved iNKRs and the MHC-X and -Y molecules deviate from the control: the distribution of X detectors to MHC-X molecules is shifted to the right (Figure 4A, black curve), indicating that X detectors evolve a high complementarity to MHC-X molecules. At the same time, the distribution of X detectors to MHC-Y molecules is shifted to the left (Figure 4A, red curve), suggesting that X detectors lose complementarity to MHC-Y molecules. Indeed, at their evolved binding threshold, X detectors recognize more than twofold more MHC-X molecules and less than half MHC-Y molecules than expected. Similarly, MHC-Y detectors evolve high complementarity to MHC-Y molecules (Figure 4B, red curve), while losing affinity to MHC-X molecules (Figure 4B, black curve).

The deviation from the expected complementarity depends on the similarity of the MHC molecules. In simulations with less similar MHC molecules (e.g., HD = 6, Figures 4C,D), the evolved receptors still specialize to their MHC molecules, albeit with lower complementarity. To quantify the difference to the expected distribution, we calculate the sum square residuals (SSR) in each simulation (Figures 4E,F). The lower the similarity of the MHC molecules, the more challenging it is for an iNKR to specialize to one MHC locus. Therefore, the complementarity distribution hardly deviates from the expected distribution with an increasing HD (e.g., HD = 8). The SSR to the control is close to zero in those simulations considering random MHC molecules.

Taken together, our results show that iNKRs evolve specificity to detect viruses downregulating particular MHC-molecules. However, the iNKRs specialization to the different MHC loci depends on the structure of the MHC molecules: if the MHC molecules within one locus share a motif and the difference between MHC loci is large, iNKRs become complementary to one MHC-I locus, while losing capacity to bind molecules from other loci.

### Similarity of the MHC molecules shapes the evolution of NKR diversity

**Figure 5:**
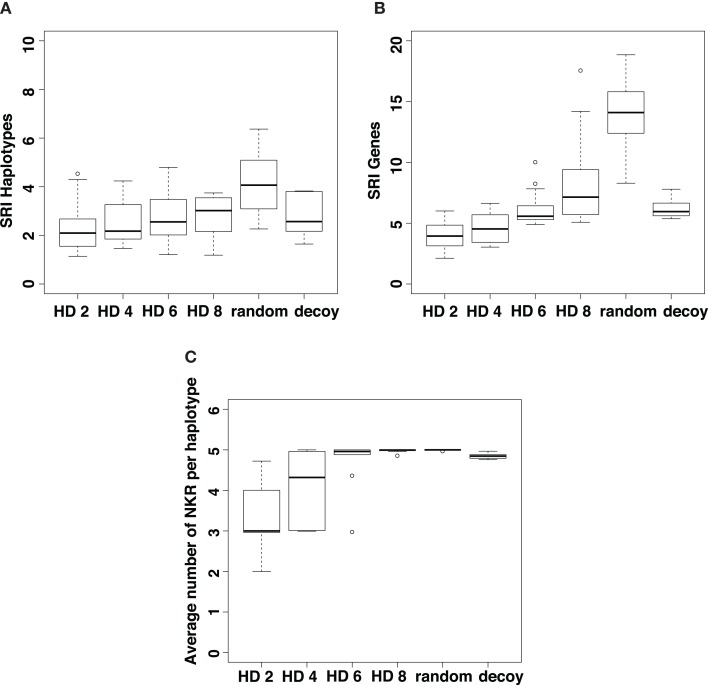
**Evolution of NKR diversity depends on the similarity of the MHC molecules**. We estimate the genetic diversity by computing the Simpson’s Reciprocal Index (SRIFL) of all haplotypes **(A)** and iNKRs **(B)** present in the population at t_end_. The SRI score is high in populations with a dissimilar MHC molecules (i.e., an increased HD) and in those populations infected with viruses evolving MHC-decoys. Additionally, we estimate the expansion of the iNKR haplotype, by measuring the number of loci per haplotype **(C)**. The selection pressure for polygenicity is smaller in populations with highly structured MHC molecules. The boxes represent the interquartile range, and the bold horizontal lines represent the median taken out of 10 simulations.

We next analyze the genetic diversity of iNKRs that evolves in our simulations. As a measure of genetic diversity, we use the Simpson’s reciprocal index (SRI, see Materials and Methods). We measure the diversity of the different NKR haplotypes (Figure 5A) and of the genes (Figure 5B) that evolve after 5 million years of evolution. Additionally, we address the evolution of polygenicity by measuring the number of loci per haplotype (Figure 5C). In all simulations, the initial haplotype encoding one gene evolves into multigene haplotypes as a result of the selection pressure exerted by the viruses. Indeed, at least two iNKRs (one binding all X molecules, and another one binding all Y molecules) are necessary for an optimal missing-self detection. In populations with highly similar MHC molecules per locus (i.e., HD = 2), these optimal X and Y detectors readily evolve, resulting in little selection for polymorphism (Figures 5A,B) and polygenicity (Figure 5C).

By contrast, the selection for diversity increases in simulations when the MHC molecules have vague motifs, making it difficult to classify them as members of the X or Y locus (e.g., HD = 8 in Figure 5). In these cases, iNKRs evolve a high specificity to detect the particular MHC-molecules that have been selectively downregulated. However, if iNKRs are very specific, they also have a high chance of not recognizing any MHC molecule in the host, and will therefore not become licensed. The chance of having at least one licensed iNKR per host (i.e., one iNKR binding to one of the four MHC-I molecules in that same host) is increased in individuals carrying several functionally different iNKRs. Therefore, heterozygote hosts carrying a multigene NKR haplotype (i.e., hosts carrying maximal 10 different genes) will have an advantage over homozygous hosts. Indeed, this heterozygote advantage in populations with dissimilar MHC molecules (i.e., HD ≥ 6) drives the evolution of a larger genetic diversity (Figures 5A,B) and polygenicity (Figure 5C).

### Selection pressure caused by selective MHC downregulation is similar to that caused by MHC-decoys

To quantify the differences in selection pressure caused by MHC decoys and selective MHC downregulation, we ran another set of simulations in which host populations are infected with viruses downregulating all MHC molecules within a host, and additionally expressing an MHC decoy (see Materials and Methods). To make a rigorous comparison, we use the same configuration as in the simulations considering selective MHC downregulation, i.e., we assume that if at least one of the licensed iNKR detects missing self (and is not “fooled” by the decoy), the host will be able to clear the infection (see Materials and Methods).

In a host population carrying random MHC molecules, the selection pressure exerted by decoys is similar to that exerted by selective MHC downregulation in populations with somewhat dissimilar MHC molecules (Figure 5). To distinguish self MHC molecules from decoys, more specific iNKRs evolve, recognizing approximately 20% of MHC molecules in the population (results not shown). Because of the higher specificity, it is more challenging for the iNKRs within a host to become licensed. Also here, the pressure to increase the licensed repertoire results in the selection for polygenicity (Figure 5C) and some polymorphism (Figures 5A,B).

## Discussion

The functional relevance of the remarkable genetic diversity of NKRs remains intriguing. In this work, we investigate the evolution of NKRs in host populations that are infected with viruses that downregulate non-overlapping subsets of MHC-I molecules. We find that this selective MHC downregulation drives the evolution of specific iNKRs, which can specialize to the different MHC loci if the MHC molecules in one locus share structural motifs.

In order to develop an insightful model of a complex evolutionary process, simplifying assumptions were necessary. Therefore, we ignore the synergy between NKRs or the direct interaction between immune cells, and focus here only on the NK mediated immune response. Furthermore, we do not model individual NK cells but consider a global NKR repertoire composed of iNKRs only. Although the role of activating receptors and their genetic diversity is equally fascinating, we did not include them in our simulations, as they are not involved in the recognition of missing-self. We also have chosen to fix the MHC polymorphism in our simulations, despite the evidence of the co-evolution between MHC class I and KIRs (29, 30). Given their different evolutionary timescales, i.e., that MHC molecules are older than both Ly49 and KIRs, we chose to model the expansion and contraction of NKR systems within an already existing MHC diversity. A fixed MHC polymorphism has also the advantage that it enables us to have a clean experiment in which we can study rigorously the evolution of iNKRs. Finally, our aim was to quantify the selection pressure of selective MHC downregulation, and therefore we have excluded from our current model other evasion mechanisms such as decoy molecules.

Several hypotheses have been proposed to describe the factors affecting the evolution of NKRs, including reproduction success (29) and viral evasion strategies (11, 12). Probably, the most intuitive explanation is that NKRs must be able to detect decreases in the expression of MHC molecules on the cell surface. We have previously shown that neither specificity nor genetic diversity are required to successfully detect missing-self (14). However, in those studies, we assumed that MHC downregulation affects all loci. In reality, not all MHC molecules are targeted equally and the challenge of NK cells might be to detect missing-self, particularly when only subsets of the MHC-I molecules are downregulated. We show that if MHC molecules within the same locus are sufficiently similar, iNKRs can easily evolve the ability to recognize them. These findings are in line with the observation that inhibiting KIRs (iKIRs) are specialized to four structural motifs on HLA molecules: A3/A11, Bw4, C1, and C2 (31). These main ligands for iKIRs are mutually exclusive, differing in unique residues that are involved in the KIR-pMHC interaction (31). While residues 77–83 define the structure of Bw4 (32–34), the dimorphism among HLA-C molecules at position 80, i.e., either asparagine or lysine, determines the classification of HLA-C1 and -C2 alleles (35, 36). Single amino acid substitutions in these key residues can have large effects on the binding to KIRs, as shown by a vast range of structural and functional studies (35, 37–39). Thus, the “micro” structures on HLA molecules clearly define a pattern for KIR recognition, and optimal detectors of these common MHC structural motifs have indeed evolved.

Our model predicts that there should be specific detectors for each locus. However, not all iKIR ligands are locus specific. The Bw4 epitope is also carried by approximately 25% of HLA-A alleles (29), hampering the discrimination between HLA-A and -B. Furthermore, the majority of HLA-A and -B alleles (approximately two-thirds in each locus) do not carry these iKIR specific epitopes, indicating that humans have no optimal HLA-A or HLA-B detectors. In contrast, the C1 and C2 motifs are carried almost exclusively by HLA-C molecules and are present in all human populations (29). The inhibiting receptors, KIR2DL1 and KIR2DL2/3, interact with either C1 or C2 (40–44), and are highly frequent in most human populations, indicating that HLA-C detectors have indeed evolved. Why only HLA-C specific detectors have evolved remains puzzling, suggesting that additional evasion mechanisms were involved in the evolution of HLA-A and B specific KIRs. A possible explanation is that viruses have evolved to downregulate only a few subsets of A and B alleles, to escape NK cell responses in some hosts, which in turn might drive the evolution of iKIRs specialized to only some subsets of MHC molecules. Investigating which viral protein targets which MHC molecule, and extending this understanding to different species would shed further light into the co-evolutionary processes between MHC-downregulating viruses and their hosts.

According to our model, the specialization to different MHC loci can exert selection pressure to evolve novel NKRs, thus providing a plausible explanation for the polygenicity and polymorphism of NKRs. However, the degree of genetic diversity that evolves in this study is somewhat limited. It could be that other immuno-evasive mechanisms, e.g., MHC-decoys, exert a stronger selection pressure on the diversification of iNKRs. We have previously shown that the selection pressure driven by viruses encoding MHC-like molecules has a much larger effect on the evolution of NKR diversity than what we report here (14, 15). This difference depends strongly on how the host’s protection is modeled, however. The high iNKR specificity that hosts required to clear decoy-encoding viruses (which in turn exerts a stronger selection pressure on the NKRs) depends strongly on whether *at least one* or *all* iNKRs in the licensed repertoire should be protective. Based on experimental evidence indicating that one interaction between an inhibiting NKR and a decoy is sufficient for the host to succumb the infection (45), we previously assumed that *all* iNKRs must be protective to clear the infection. However, it is counterintuitive to have one inhibiting interaction dominating the host’s NK cell response. Theoretically, if one iNKR is able to detect missing-self (i.e., to be protective), the NK cell subsets carrying that receptor should proliferate and provide some degree of protection. Therefore, we adopted a more natural assumption in our current model for selective downregulation, i.e., that a host needs *at least one* protective iNKR to clear the infection. The actual protection might lie between *all* and *at least one* protective iNKRs, but the current understanding of the contribution of each NK cell subset to host’s protection during a viral infection remains limited. To mechanistically understand the data (45), the elucidation of how many iNKRs are required for protection is absolutely necessary. Nevertheless, we here show that both immunoevasive mechanisms affect the diversification of iNKRs.

Summarizing, we have shown that selective MHC downregulation can drive the evolution of specific NKRs. However, the evolution of optimal MHC-loci detectors does not require an extensive degree of genetic diversity. Therefore, selective MHC downregulation is unlikely to be the only explanation underlying the extensive genetic diversity observed in the NKR families.

This suggests that the evolution of NKRs is subject to several viral immunoevasive mechanisms and that all of these contribute in different degrees to the evolution of polymorphic, polygenic, and specific NKR genes.

## Materials and Methods

### Agent based model

The ABM consists of two types of agents, i.e., hosts and viruses, and three types of events: birth, death, and infection. During each time step of 1 week, we screen all hosts in a random order and confront them to one of the events. Hosts age over time and their ages, infection states, and infection types are updated at the end of every time step. This cycle is repeated for 1 million years to simulate long term evolution.

The model used here is based on our previously published ABM (15). Briefly, we model a host population consisting of simplified humans infected with non-lethal viruses causing chronic infections. The hosts are diploid, carrying two polymorphic MHC loci and an NKR cluster, which are encoded on different chromosomes. Here, we only model the evolution of inhibiting NKRs, as activating NKRs are not involved in missing-self detection. We use bit strings to model iNKRs and MHC molecules as a simplified representation of amino acids. To each iNKR, a random binding threshold L between 1 and 16 is assigned. Molecules are only allowed to interact if the longest adjacent complementary match L_c_ between the strings exceeds this binding threshold L (Figure S1 in Supplementary Material). Thus, the binding threshold (L) and the complementarity between bit strings (L_c_) determine the specificity of each receptor. In the following sections, we provide a detailed description of the differences between the current model and our previously published models (14, 15). All model parameters are fully described in Table 1.

**Table 1:** **Parameters of the agent-based model**.

Parameter	Value
Time step	1 week
Simulation time	5 million years
**Host parameters^a^**	
Maximal population size *N*_max_	5000 individuals
MHC diversity	2 loci, each with 15 alleles
Maximal number of NKR loci per haplotype	5
Bit string length	16 bits^b^
Host mutation rate *μ* (i.e., point mutation)	0.00005 per gene per birth event
Probability of generating a random novel NKR	0.1 per mutation event
**Infection^c^**	
Infection state *i*	1 (acute), 2 (chronic)
Effect of viral load on the death rate VL_i_	0.1 (for *i* = 1), 0.06 (for *i* = 2) per year
Probability of viral transmission during acute phase p_ac_	0.85 per contact
Probability of viral transmission during chronic phase p_ch_	0.15 per contac
Probability of clearing the infection p_cl_	0 if missing-self is not detected, or 0.6 if missing self is detected
Immunity time t_i_	10 years
Acute infection time t_inf_	4 weeks
**Initial conditions**	
Initial population size N_init_	4500 individuals with homozygous hosts, encoding two copies of the same NKR in their gene cluster)

*^a^The death and birth rate parameters are age-dependent and have been chosen according to a human population (46). For a full description of the age-dependency of birth and death rate, see Ref. (14)*.

*^b^By using 16-bit strings, a large enough theoretical repertoire of 65,536 sequences is represented*.

*^c^We choose the parameters used for the infection such that the epidemic can be maintained. Changing the length of the acute phase or the probabilities of clearance do not affect our results on the evolution of the NKRs qualitatively (results not shown)*.

### MHC molecules

We create two gene pools of 15 MHC molecules, each which reflects the most common HLA B and C alleles in the European population (47). We perform simulations differing in the similarity of their MHC molecules. In the structured simulations, MHC molecules can have a hamming distance (HD) of maximal 2, 4, 6, or 8 bits to each other. To create the MHC molecules with HD = 2, we first create one random bit string and generate all possible (16) strings that have a HD = 1 to the original sequence. We take all of these strings and fill the pool of MHC-X molecules. The pool for the Y molecules is filled similarly, yet with a different randomly generated bit string (i.e., the expected HD between X and Y alleles is 8 bits). For the populations with MHC molecules with HD = 4, we repeat this procedure: for each of the “first generation mutants” (i.e., those with HD = 1 to the original, HD = 2 to each other), we generate again all possible strings that have a HD = 1. This ensures that the HD between the strings in the second generation is maximally 4. We randomly select 15 strings out of these “second-generation mutants” to fill one MHC pool. Accordingly, we chose 15 strings from the “third-generation mutants,” and from the “fourth-generation mutants” to fill the MHC pools with HD = 6 and HD = 8, respectively. For the simulations with random MHC molecules, each gene pool is filled with 15 randomly generated bit strings. We also use random MHC molecules in the simulations considering MHC-decoys.

### NKR molecules

During the hosts’ sexual reproduction, iNKR mutate with a rate *μ* (see Table 1). In every mutation event, NKRs undergo point mutation and their L can increase or decrease by one, thereby simulating a gradual and slow mutation process. To decrease computational time, we additionally allow for the random generation of a novel bit string with a probability *p* = 0.1 within each mutation event, and assign a random value 1 ≤ L ≤ 16. Receptors with L larger than 13 will typically not recognize any MHC molecules in the population, and are therefore considered to be non-functional. We refer to these non-functional NKRs as *pseudo genes*. This method allows us to model the contraction and expansion of the NKR gene complex, as haplotypes containing pseudo genes are effectively shorter than those composed of fully functional NKRs.

### Viral infections: Selective MHC downregulation

In these simulations, we consider two viral species. Both species come with a particular viral load, which is implemented as an increase of the hosts’s death rate, VL_i_, depending on the infection state i (see Table 1). Both species, A and B, downregulate the MHC expression in the infected host, but species A downregulates the molecules in the MHC-X locus, whereas species B downregulates those in the Y locus. Viruses do not evolve in this model. The infection will be cleared with a probability p_cl_ depending on the interactions of the licensed iNKRs with the expressed MHC-I molecules. Importantly, we do not model T cells explicitly, but these are implicitly taken into account via the probability of clearing the infection p_cl_. We basically assume that if a virus downregulates MHC-X and/or MHC-Y, it will escape the T cell responses restricted to X or Y, and we model the lack of T cell responses by lowering p_cl_ to 0. However, if the NK cells in that host are able to detect missing self, the host will have a higher protection. If a virus downregulates all MHC-A molecules, and at least one licensed iNKR binds none of the MHC-B molecules (i.e., if there is *at least one* protective iNKR), the NK cell subset carrying that receptor will perceive a reduced inhibiting signal, resulting in a successful missing-self detection. In this case, the infection is cleared with p_cl_ = 0.6 (see Figure 1).

### Viral infections: MHC decoys

In these simulations, we reconsider our previous study with several viruses expressing MHC decoys (14, 15). A decoy virus downregulates the expression of all MHC molecules in that host and encodes one MHC-like molecule. The evolution of decoy molecules is modeled by allowing the virus to adopt a randomly selected MHC molecule from its host. Because we fix the MHC polymorphism to 15 alleles per locus, the maximal number of decoy proteins that can arise in the population is 30. Each virus comes with a viral load, which is implemented by increasing the host’s death rate, VL_i_ depending on the infection state i (see Table 1).

To make a fair comparison with the downregulation model, we adopt the same rule of protection here (and not use the previous assumptions as in (14, 15)). We consider different levels of protection against a decoy virus, depending on the success of the virus to escape the NK cell response. If one of the licensed iNKR is not “fooled” by thedecoy molecule (i.e., if there is *at least one* protective iNKR), the NK cell subsets carrying that receptor will successfully detect “missing-self” and the host will clear the infection with a probability p_cl_ = 0.6. In contrast, if none of the licensed iNKRs detects missing-self, the decoy virus will be successful. We model the immune escape by setting the probability of clearing the infection to 0, letting the host become chronically infected.

### Model initialization

The model is initialized with a host population of 4500 individuals, with random ages between 10 and 70 years. Hosts carry two MHC loci, each of them encoding two genes from the MHC-X gene pool, and two genes from MHC-Y pool, respectively. The initial host population is homozygous for NKRs, carrying a NKR haplotype composed of two copies of one degenerate iNKR, i.e., an iNKR being able to recognize all MHCs in the population.

### Clustering of NKRs

We create a (*n* × *m*) matrix *A* to describe the binding of all *n* iNKRs and *m* MHC-I molecules in the population. If the *i*-th iNKR binds the *j*-th MHC-I molecule, the entry of the matrix *A_ij_* will be 1, otherwise *A_ij_* will be 0. We cluster the iNKRs and the MHC-I molecules according to this binding matrix by clustering both rows and columns. We use the Manhattan measure of distance and the Ward clustering algorithm.

### Genetic diversity

The Simpson’s Index is a measurement of diversity that can be interpreted as the probability that two randomly chosen molecules from two random hosts in the population are identical. The lower the Simpson’s Index, the higher is the diversity of molecules in the population. The reciprocal of the Simpson’s Index (SRI) defines a “weighted diversity” (48), which was calculated as follows: $\mathrm{SRI}=\frac{1}{\sum_{i=1}^{N}f_{i}^{2}}$, where *f_i_* is the fraction of the molecule *i* over all NKRs in the population, and *N* is the total number of unique NKRs. The higher the SRI, the higher the diversity of NKR genes in the population.

### Analysis of recognized MHC molecules

To determine the expected distribution of the maximal complementary match L_c_, we generate 1000 random NKRs and measure their longest complementary adjacent match to randomly generated MHC molecules. The frequency of recognized MHCs with a particular L_c_ is averaged over 1000 of these “experiments.”

To analyze how this distribution changes after evolution, we measure the longest complementary adjacent matches between iNKRs and the MHC-A, and MHC-B molecules after 1 million years of evolution. An iNKR is classified as an MHC-X detector if it binds to more MHC-X molecules than MHC-Y molecules at its evolved L. Similarly, MHC-Y detectors are those iNKRs that recognize a larger number of Y molecules than X molecules. The rare set of iNKRs recognizing the same number of X and Y MHC molecules are classified as neutral receptors and are excluded from this analysis. The final distribution is the average out of 10 simulations.

We also analyze the distribution of L_c_ between the evolved iNKRs and random MHC molecules by measuring the L_c_ of each iNKR to random MHC molecules, and repeating this experiment 1000 times. This frequency distribution does not deviate from the expected values (results not shown).

## Conflict of Interest Statement

The authors declare that the research was conducted in the absence of any commercial or financial relationships that could be construed as a potential conflict of interest.

## Supplementary Material

The Supplementary Material for this article can be found online at http://journal.frontiersin.org/article/10.3389/fimmu.2015.00311
