# Supplementary material for: Can Selective MHC Downregulation Explain the Specificity and Genetic Diversity of NK Cell Receptors?
Source: Front Immunol. 2015 Jun 16;6:311. doi: 10.3389/fimmu.2015.00311 (PMC4468891; doi:10.3389/fimmu.2015.00311)
Supplement: Supplementary file 2 [file Image_1.PDF]

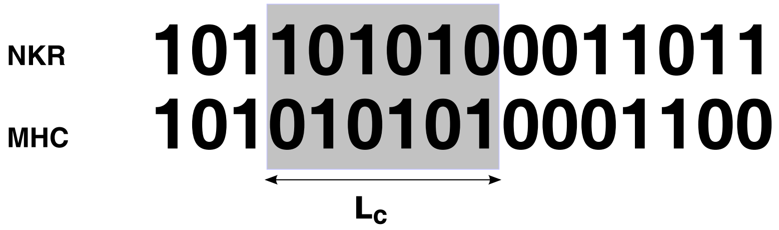

Figure S1. Schematic representation of the molecular interactions in our model. If the longest complementary adjacent match (L_c) between two strings exceeds a threshold L, the molecules interact. In this example, the NKR will interact with the MHC molecule if its $L \geq 6$.
